# Body Composition Changes in Children during Treatment for Moderate Acute Malnutrition: Findings from a 4-Arm Cluster-Randomized Trial in Sierra Leone

**DOI:** 10.1093/jn/nxab080

**Published:** 2021-04-20

**Authors:** Devika J Suri, Isabel Potani, Akriti Singh, Stacy Griswold, William W Wong, Breanne Langlois, Ye Shen, Kwan Ho Kenneth Chui, Irwin H Rosenberg, Patrick Webb, Beatrice L Rogers

**Affiliations:** Friedman School of Nutrition Science and Policy, Tufts University, Medford, MA, USA; Friedman School of Nutrition Science and Policy, Tufts University, Medford, MA, USA; Department of Nutritional Sciences, Faculty of Medicine, University of Toronto, Toronto, Ontario, Canada; Translational Medicine Program, Hospital for Sick Children, Toronto, Ontario, Canada; Friedman School of Nutrition Science and Policy, Tufts University, Medford, MA, USA; Friedman School of Nutrition Science and Policy, Tufts University, Medford, MA, USA; USDA/ARS Children's Nutrition Research Center, Department of Pediatrics, Baylor College of Medicine, Houston, TX, USA; Friedman School of Nutrition Science and Policy, Tufts University, Medford, MA, USA; Friedman School of Nutrition Science and Policy, Tufts University, Medford, MA, USA; School of Medicine and Public Health, Tufts University, Medford, MA, USA; Friedman School of Nutrition Science and Policy, Tufts University, Medford, MA, USA; Friedman School of Nutrition Science and Policy, Tufts University, Medford, MA, USA; Friedman School of Nutrition Science and Policy, Tufts University, Medford, MA, USA

**Keywords:** moderate acute malnutrition, preschool children, Sierra Leone, body composition, deuterium dilution, supplementary nutritious foods, corn-soy blend, ready-to-use supplementary food, relapse

## Abstract

**Background:**

Measures that better describe “healthy” and sustainable recovery during nutritional treatment of children with moderate acute malnutrition (MAM) are needed.

**Objectives:**

We compared changes to body composition among children receiving 1 of 4 specialized nutritious food (SNFs) during treatment of MAM and by recovery and relapse outcomes.

**Methods:**

The study was nested within a prospective, cluster-randomized, community-based, cost-effectiveness trial assessing 4 SNFs to treat children aged 6–59 mo with MAM [midupper arm circumference (MUAC) ≥11.5 cm and <12.5 cm without bipedal edema] in Sierra Leone. Biweekly SNF rations (1 of 3 fortified-blended foods or a lipid-based nutrient supplement) were given until children recovered (MUAC ≥12.5 cm), or up to 7 rations (∼12 wk). Deuterium dilution was used to estimate fat-free mass (FFM) and fat mass (FM) at enrollment and after 4 wk of treatment to ensure similar treatment exposure among the participants. Another MUAC measurement was performed among recovered children 4 wk after program exit to determine whether recovery was sustained. ANOVA, paired *t* tests, and linear regression models were used to determine significant differences in changes from baseline to 4 wk.

**Results:**

Among 312 analyzed participants, mean baseline weight comprised ∼80% FFM; mean weight gained after 4 wk comprised ∼82% FFM. Changes in FM and FFM among 4 SNFs were similar. Children who recovered gained more weight (241%), FFM (179%), and weight-for-height *z* score (0.44 compared with 0) compared with those who did not recover; sustainers gained 150% more weight. FM gains were positive among recovered children and sustainers, as well as negative among those who did not recover or sustain recovery, but not significantly different.

**Conclusions:**

Four SNFs had similar effects on body composition in children after 4 wk of treatment for MAM, showing a healthy pattern of weight gain, the majority being FFM. Differential responses to treatment underscore a need for further research to provide targets for healthy, sustainable recovery. This trial was registered at clinicaltrials.gov as NCT03146897.

## Introduction

There is growing recognition that supplementary feeding programs to treat moderate acute malnutrition (MAM) in children should assess outcomes that complement and expand on conventional anthropometric indicators, such as midupper arm circumference (MUAC) and weight-for-height *z* score (WHZ) (1–3). The latter anthropometric measures do not reflect other important aspects of child development such as cognition, immune function, and long-term health outcomes (4). In other words, we need a better understanding of and indicators to measure what constitutes “healthy” recovery from MAM (5) in order to inform development of more effective programming and improved short- and long-term health outcomes for malnourished children.

Body composition is one such potential indicator; it measures the proportion of fat mass (FM) compared with fat-free mass (FFM) in the body, which has been shown to be important for short-term survival in both healthy and sick children (6). Evidence also suggests that body composition in infancy can presage adult noncommunicable disease risk; in particular, rapid and/or catch-up weight gain in early childhood has been associated with adiposity, insulin resistance, obesity, and noncommunicable diseases later in life (7, 8). Currently, guidelines for absolute or proportional gain of FFM and FM in children while recovering from MAM are limited (7, 9). Furthermore, there is debate around whether different compositions of specialized nutritious foods (SNFs)—particularly between fortified blended foods (FBFs) and lipid-based nutrient supplements (LNSs)—affect body composition and whether that has consequences for the short-term sustainability of the recovery from MAM, as well as long-term health and disease risks (10–12).

Only a few studies have examined body composition in the context of treatment of MAM, and to our knowledge, no studies have looked at the relation between body composition changes during treatment for MAM and short-term relapse postrecovery. Given the concern for long-term implications of childhood MAM and treatment with SNFs, more data on the changes in body composition during SNF treatment for MAM are needed to help inform programs that are effective in addressing both short- and long-term health outcomes.

In order to examine the effects of 4 SNFs (including 3 FBFs and 1 LNS) on changes in body composition, we conducted a study among a subgroup of children enrolled in a MAM treatment trial in Sierra Leone. We compared body composition with the conventional anthropometric measures linked to MAM recovery and to the sustainability of that recovery posttreatment (i.e., relapse). We aimed to determine whether changes in FM and FFM would help to discriminate among responses to the different SNFs and whether body composition measures would add meaningfully to the assessment and understanding of “healthy” recovery beyond the anthropometric measures.

## Materials and Methods

### Study design

This study was nested within a prospective, cluster-randomized, controlled clinical and cost-effectiveness trial assessing 4 SNFs to treat children aged 6–59 mo with MAM, defined as MUAC ≥11.5 cm and <12.5 cm without bipedal edema, in Pujehun district, Sierra Leone, from April 2017 to November 2018 (registered at clinicaltrials.gov as NCT03146897). Participants in this community-based intervention included eligible children screened at 29 participating peripheral health units (PHUs) that were randomized to treat MAM with 1 of 4 SNFs: corn-soy blend plus (CSB+) with oil, super cereal plus amylase (SC + A), corn-soy-whey blend with oil, and ready-to-use-supplementary food (RUSF). Participants were given biweekly rations until they “recovered,” defined as achieving a MUAC ≥12.5 cm, at which point they were given a final ration and discharged. Other possible outcomes included children who deteriorated to severe acute malnutrition (SAM), died, defaulted (i.e., were lost-to-follow-up), or were considered “failed” if they received up to 7 rations (∼12 wk) of treatment and remained MAM. More details on the main study protocol and results can be found elsewhere (13).

### Participants and sample size

Participants were recruited from 8 PHUs (2 per arm), selection of which was purposively based on logistical needs and on the number of MAM cases, with a target maximum sample size of 200 per study arm within the allocated data collection time. All participants (beneficiary children and their caregivers) at the selected PHUs were eligible to enroll in the study. Accounting for clustering (using a moderate design effect of 1.115, as low intraclass correlation was expected), stratified analyses, and loss to follow-up, a sample size of 89 per group would achieve 80% power to detect a mean of paired differences of a 0.2 percentage point change in FFM with an estimated standard deviation of differences of 0.5 at a 0.05 significance level.

### Ethical approval

This study was approved by the Tufts University Health Sciences Institutional Review Board and Sierra Leone Ethics and Scientific Review Committee. Written informed consent was obtained from caregivers of all participants.

### Deuterium dilution method

The deuterium dilution (DD) technique was used to assess body composition. This technique is based on a 2-component model of body composition (14) and has been used previously to assess body composition in children with MAM (15). Participants ingest an oral dose of deuterium oxide (D_2_O); urine is subsequently collected at least 4 h postdose to allow sufficient time for the isotope to reach isotopic equilibrium. For this study, recruited participants were asked to return to the clinic the day after they were enrolled in the main study to ensure there was sufficient time to complete the DD protocol. Participants were again asked to return to the clinic for a follow-up body composition analysis on the day after receiving their fourth week of treatment. This 4-wk time period was chosen so that all enrolled children would have the same exposure to the intervention, as children who graduated from the MAM treatment program would not continue to receive the treatment food (those who graduated at 2 wk would receive 1 more food ration, which would bring them to a total of 4 wk on the study food).

Diluted D_2_O was prepared using a 1:10 dilution of a 99% D_2_O (Sigma Aldrich) with locally purchased bottled water. Each dilution was ∼1000 mL and was made of ∼905 g of bottled drinking water and 90 g of D_2_O. As samples containing deuterium are susceptible to contamination, which can result in over- or underestimation of body composition values, the first 2 wk of the study were considered a pilot test of the DD technique under the field conditions in Pujehun. In addition, samples of the dose, water, and urine for 16 study participants from 7 sites were sent for laboratory analysis, and plausibility of the findings was confirmed.

At baseline, participants were given a dose of diluted D_2_O at 1 g/kg body weight. At end time (4 wk), the dose was doubled to *1*) minimize natural variation in isotope enrichment in the postdose urine sample and *2*) offset the effect of residual deuterium from the first dose on the deuterium enrichment at the 4-wk assessment. The doses were given orally using locally purchased syringes, and participants were not required to fast at any time before dose administration. Spills were recovered with preweighed cotton balls. Losses were estimated from the difference in weights of the cotton balls before and after collecting the spill. When spills could not be successfully collected, this was noted in the participant's record. Other events that required noting on the day of D_2_O dosing were vomiting, diarrhea, and any other occurrence that the study personnel thought might affect the outcome of the study.

After D_2_O administration was complete, participants were not allowed to take any food or drink for 2 h, after which they were allowed to eat and drink water and breastmilk under the observation of study personnel. Although free intake of water was permitted, the amount of breastmilk taken was estimated by weighing children before and after breastfeeding. Body water calculated from the DD method was adjusted for water coming from breastmilk consumed during the 4-h protocol, assuming breastmilk has a water content of 87%. Metabolic water generated from nonaqueous food consumption during the 4-h period was considered negligible and was not adjusted.

Urine samples were collected before dosing with D_2_O and after a minimum of 4 h following D_2_O administration (16), allowing the isotope to reach isotopic equilibrium. Urine was collected using PDC Healthcare–Assure Urine Collectors urine bags (ThermoFisher Scientific). Aliquots of ∼1.5 mL of baseline and postdose urine samples were transferred to airtight cryovials from the urine collection bags and then placed in cryoboxes; this was done at a urine collection station. The cryoboxes were stored and transported in an ice box to the study office for storage in a freezer at –20°C for at ∼4 wk, after which they were transferred to the University of Makeni Infectious Disease Research Laboratory, which was 8 h away, and kept in a –20°C freezer until shipment to the USDA/Agricultural Research Service Children's Nutrition Research Center at Baylor College of Medicine in Houston, Texas, for isotope ratio analyses. Refrigeration and freezing are not required but recommended for samples using the DD technique, due to potential mold and bacteria growth, which would result in dilution of the isotopes in the urine samples (17).

The deuterium content of urine samples, the diluted dose, and water used to prepare it were analyzed in duplicate at the Baylor laboratory using continuous-flow isotope ratio mass spectrometry using a method described by Wong and Clarke (18). FFM in kilograms was calculated from total body water (TBW) measured using the DD method after correcting for isotope sequestration in nonaqueous tissues (19) and using age- and sex-specific appropriate hydration factors (proportion of water in FFM, H) (20): FFM = TBW/H. FM in kilograms was then calculated as the difference between body weight (W) and FFM: FM = W – FFM. The best-fit equations to calculate the hydration factors based on age (mo) were as follows:

(1) $$\begin{eqnarray*}
\rm {Girls: H} &=& \big(- {\rm 0.0003} \times {\rm Age}^{\rm 3} + {\rm 0.027} \times {\rm Age}^{\rm 2} - {\rm 0.704}\nonumber\\
&&\times \,{\rm Age} + 84.064 \big)\big/\rm 100.
\end{eqnarray*}$$

(2) $$\begin{eqnarray*}
{\rm Boys: H} &=& \big( - 0.001 \times {\rm Age}^{3} + 0.037 \times {\rm Age}^{2} - {0.710}\nonumber\\
&&\times \,{\rm Age} + 83.764 \big)\big/{100}.
\end{eqnarray*}$$

The hydration factors might not be most appropriate for the malnourished children. However, they were applied uniformly to the malnourished children receiving the 4 SNFs. Therefore, the hydration factors should not affect the comparison of the effects of the 4 SNFs on changes in body composition and recovery.

### Statistical analysis

Outcomes of interest included changes in FFM, FM, FFM % (FFM/body weight × 100), and FFM index (FFM/height^2^) after 4 wk of treatment. Demographic and anthropometric data were collected by the Four Foods MAM Treatment Study; variables used for this analysis included weight in kilograms, MUAC, WHZ, age at enrollment, sex, a wealth scale (13), whether the child started the MAM treatment program from SAM treatment, and whether the child recovered from MAM within 12 wk. Unphysiologic TBW values due to errors in dose weight and dose spillage (>10%) were excluded from the data set.

Participants’ baseline characteristics, pooled and by study arms, were tabulated. Overall descriptive statistics for baseline, 4 wk, and changes in anthropometry and body composition variables over 4 wk were calculated, and paired *t* tests were used to determine significant differences between baseline and 4-wk values. Changes in anthropometry and body composition over the 4 wk of treatment were compared among the 4 study arms by outcome (recovered, developed SAM, or failed) and sustained recovery outcomes (MUAC ≥12.5 cm at 4 wk after program exit or relapsed to MUAC <12.5 cm) using ANOVA. To compare among study arms, linear regression models (Wald test) were also used to control for baseline characteristics and potential confounders. Initially, mixed models were used to adjust for clustering at the PHU levels, but due to the negligible intraclass correlation effect, the results essentially mirrored that of the linear regression, and thus the simpler linear models were reported. Models were evaluated for goodness of fit, collinearity, and influential outliers. Data management and analysis were conducted using Excel 2017 (Microsoft) and Stata 15 (StataCorp).

## Results

**FIGURE 1:**
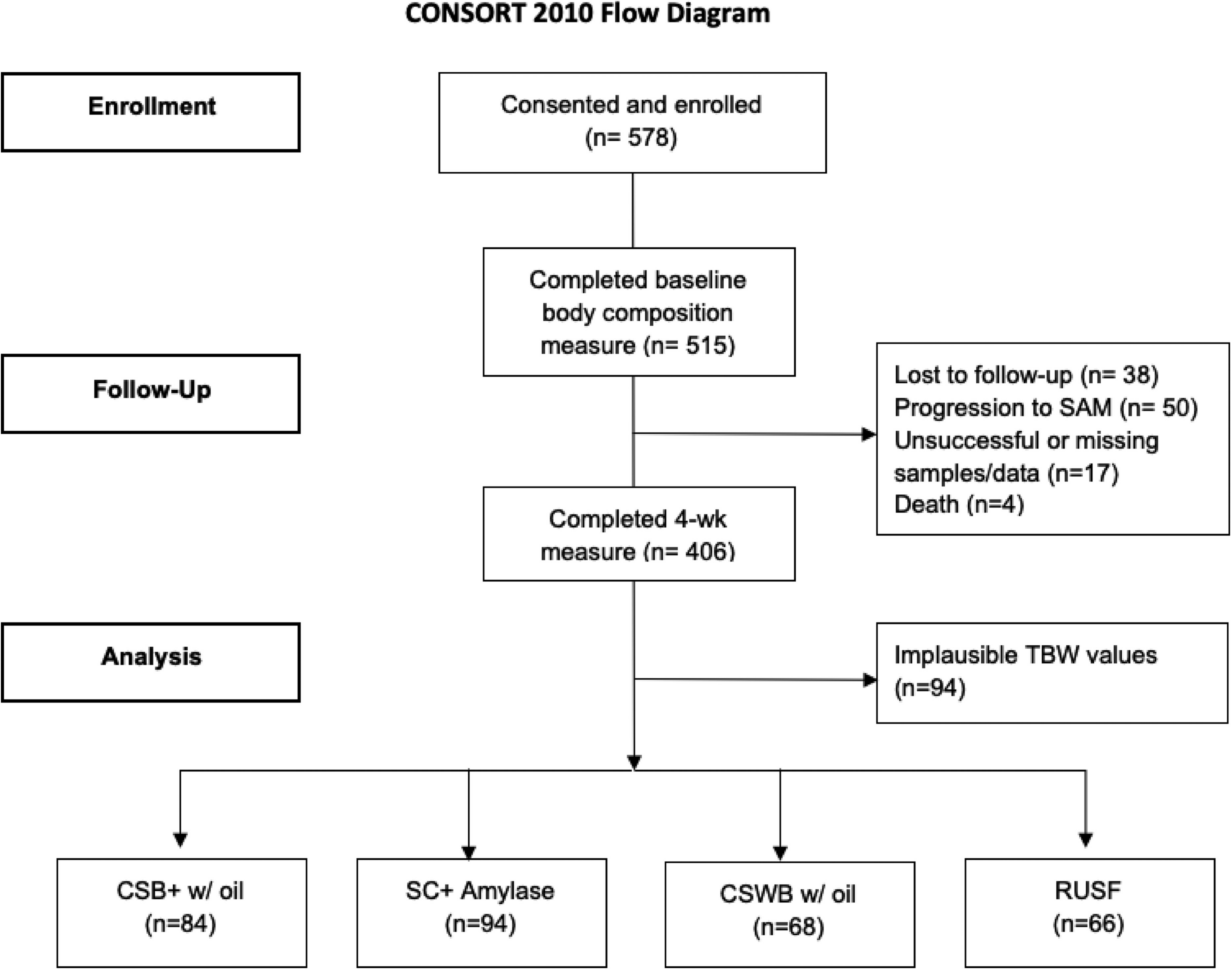
Flow diagram of participants through the study. CSB+, corn-soy blend plus; CSWB, corn-soy-whey blend; RUSF, ready-to-use supplementary food; SAM, severe acute malnutrition; SC+, super cereal plus; TBW, total body water.

A total of 578 children were recruited. Of these, 515 (89.1%) children completed the baseline deuterium dilution procedure, and 406 (70.2%) completed the 4-wk follow-up procedure (**Figure 1**). Reasons for not completing the 4-wk measure included loss to follow-up (38), progression to SAM (50), unsuccessful or missing samples or other data collection issues (17), and death (4). An additional 94 participants were removed from the analysis data set due to unphysiologic TBW values stemming from methodologic issues, including errors in dose weight and dose spillage. A total of 312 participants were included in the final analysis, as per their originally assigned groups. The participants who were dropped differed from the analyzed participants by sex, baseline MUAC, and whether they started from SAM treatment (**Supplemental Table 1**).

**Table 1** shows baseline descriptive statistics of the analyzed sample. Because of the nature of recruitment for this study (from 2 of 7 PHUs per arm), the 4 study arms were not necessarily expected to have comparable characteristics; however, we found that they were similar with the exception of wealth quintile distribution. As such, this variable was controlled for in the adjusted models. Rates of recovery and 4-wk sustained recovery were similar among the study arms. At baseline, participants weighed an average of 6.53 kg, of which 5.24 kg was FFM (80.1%) and 1.29 kg was FM (**Table 2**). Over 4 wk of treatment for MAM, participants gained an average of 0.45 kg, of which 0.37 kg was FFM (∼82%) and 0.07 kg was FM; that is, a slightly higher proportion of FFM was gained compared with starting body composition, but on average, the change in FFM % (0.26 percentage points) from baseline to 4 wk was not significant (*P* = 0.589).

**TABLE 1:** Baseline characteristics and recovery outcomes of children included in analysis, overall and by study arm^1^

Characteristic	Total	CSB+ w/oil	SC + A	CSWB w/oil	RUSF
*N*	312	84	94	68	66
Age, mo	12.2 ± 7.18	12.0 ± 6.64	12.7 ± 7.80	12.5 ± 7.20	11.5 ± 6.97
Females	173 (55)	49 (58)	55 (59)	35 (51)	34 (52)
Previous SAM	78 (25)	23 (28)	23 (24)	15 (22)	17 (26)
HAZ	–2.72 ± 1.15	–2.73 ± 1.13	–2.73 ± 1.04	–2.84 ± 1.26	–2.58 ± 1.19
WHZ	–1.70 ± 0.74	–1.63 ± 0.69	–1.76 ± 0.74	–1.85 ± 0.76	–1.57 ± 0.75
Wealth quintile^2^					
Lowest	69 (22)	22 (26)	11 (12)	17 (25)	19 (29)
Low	52 (17)	9 (11)	13 (14)	9 (13)	21 (32)
Middle	62 (20)	16 (19)	23 (25)	10 (15)	13 (20)
High	71 (23)	20 (24)	21 (23)	18 (26)	12 (18)
Highest	56 (18)	17 (20)	24 (26)	14 (21)	1 (2)
Recovered within 12 weeks	229 (73)	61 (73)	70 (74)	51 (75)	47 (71)
Sustained recovery for 1 mo^3^	169 (77)	46 (78)	55 (81)	37 (79)	31 (69)

1 Values are presented as mean ± SD or number (%). CSB+, corn-soy blend plus; CSWB, corn-soy-whey blend; HAZ, height-for-age *z* score; RUSF, ready-to-use supplementary food; SAM, severe acute malnutrition; SC + A, super cereal plus with amylase; WHZ, weight-for-height *z* score.

2 Significantly different among groups (*P* < 0.001, χ^2^ test); CSB+ w/oil (*P* = 0.001), SC + A (*P* < 0.001), and CSWB w/oil (*P* = 0.001) significantly different from RUSF (between group χ^2^ test).

3 Ten recovered children missing follow-up data*: n* = 59 (CSB+ w/oil), *n* = 68 (SC + A), *n* = 47 (CSWB), and *n* = 45 (RUSF).

**TABLE 2:** Anthropometry and body composition over 4 wk of treatment for MAM in analyzed participants (*n* = 312)^1^

Characteristic	Baseline	4 wk	Change	*P* value^2^
Weight, kg	6.53 ± 0.96	6.98 ± 1.01	0.45 ± 0.39	<0.001
WHZ^3^	–1.70 ± 0.74	–1.41 ± 0.82	0.30 ± 0.61	<0.001
MUAC, cm^2^	12.0 ± 0.27	12.2 ± 0.44	0.32 ± 0.46	<0.001
Total body water, kg	4.16 ± 0.71	4.44 ± 0.74	0.29 ± 0.45	<0.001
FFM, kg	5.24 ± 0.95	5.61 ± 0.98	0.37 ± 0.57	<0.001
FM, kg	1.29 ± 0.48	1.37 ± 0.49	0.07 ± 0.60	0.037
FFM %	80.1 ± 7.0	80.3 ± 6.8	0.26 ± 8.4	0.589
FFM index^3^	11.6 ± 1.2	12.0 ± 1.1	0.49 ± 1.3	<0.001

1 Values are presented as mean ± SD. FFM, fat-free mass; FM, fat mass; MAM, moderate acute malnutrition; MUAC, midupper arm circumference; WHZ, weight-for-height *z* score.

2 Paired *t* tests used to compare baseline compared with 4-wk measure.

3 At 4 wk, *n* = 225, WHZ; *n* = 218, MUAC; *n* = 205, FFM index.

**FIGURE 2:**
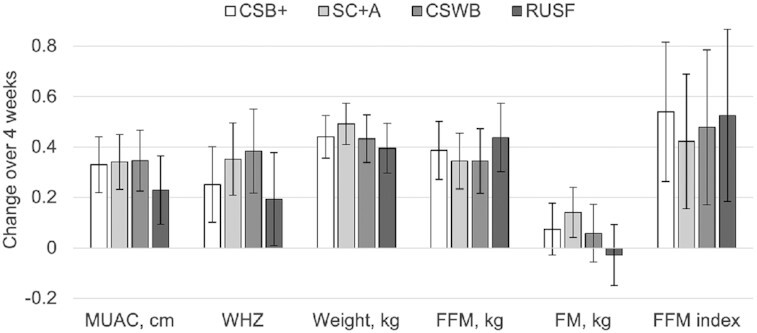
Comparison of adjusted mean changes to body composition and anthropometric indicators in children after 4 wk of treatment for MAM among 4 study arms. Error bars represent 95% confidence intervals. No significant differences by indicator among study arms based on linear regression models adjusted for baseline measure, age, sex and wealth quintile. *n* = 218, MUAC; *n* = 225, WHZ; *n* = 312, weight; *n* = 311, FFM; *n* = 311, FM; *n* = 205, FFM index. CSB+, corn-soy blend plus; CSWB, corn-soy-whey blend; FFM, fat-free mass; FM, fat mass; MAM, moderate acute malnutrition; MUAC, midupper arm circumference; RUSF, ready-to-use supplementary food; SC + A, super cereal plus amylase; WHZ, weight-for-height *z* score.

There were no statistically detectable differences in anthropometry or body composition changes among the 4 study arms after 4 wk of treatment (**Table 3**). These findings were consistent in the regression models (**Figure 2**), which were adjusted for age, sex, and wealth quintile. Trends in changes to anthropometry and body composition were fairly similar among the groups, with the exception of the proportions of FFM to FM, which appeared to vary among the groups: on average, children in SC + A gained the most FM and thus lost the most FFM %, while children in RUSF gained the least FM and thus had the highest increase in FFM % over the study, but these variations were not statistically different.

**TABLE 3:** Baseline values and changes in anthropometry and body composition after 4 wk of treatment for MAM in analyzed participants, by study arm (*n* = 312)^1^

Characteristic	CSB+ w/oil	SC + A	CSWB w/oil	RUSF	*P* value^2^
*N*	84	94	68	66	
Baseline					
Weight, kg	6.55 ± 1.09	6.57 ± 0.98	6.52 ± 0.78	6.47 ± 0.95	0.915
WHZ	–1.63 ± 0.69	–1.76 ± 0.74	–1.85 ± 0.76	–1.57 ± 0.75	0.105
MUAC, cm	12.0 ± 0.27	12.0 ± 0.26	11.9 ± 0.27	11.9 ± 0.28	0.264
Total body water, kg	4.15 ± 0.64	4.19 ± 0.81	4.16 ± 0.65	4.11 ± 0.72	0.914
FFM, kg	5.23 ± 0.86	5.29 ± 1.07	5.25 ± 0.87	5.18 ± 0.96	0.901
FM, kg	1.33 ± 0.52	1.28 ± 0.49	1.27 ± 0.49	1.29 ± 0.43	0.871
FFM %	80.0 ± 6.39	80.2 ± 7.48	80.4 ± 7.57	79.8 ± 6.71	0.968
FFM index	11.6 ± 1.18	11.6 ± 1.20	11.6 ± 1.16	11.7 ± 1.04	0.968
Change after 4 wk^3^					
Weight, kg	0.43 ± 0.39	0.48 ± 0.41	0.44 ± 0.38	0.41 ± 0.38	0.696
WHZ	0.24 ± 0.57	0.35 ± 0.57	0.42 ± 0.65	0.16 ± 0.64	0.154
MUAC, cm	0.29 ± 0.49	0.32 ± 0.41	0.37 ± 0.49	0.30 ± 0.48	0.827
Total body water, kg	0.29 ± 0.43	0.26 ± 0.51	0.28 ± 0.45	0.33 ± 0.39	0.791
FFM, kg	0.38 ± 0.54	0.33 ± 0.64	0.37 ± 0.57	0.42 ± 0.49	0.807
FM, kg	0.05 ± 0.59	0.15 ± 0.66	0.07 ± 0.63	–0.01 ± 0.49	0.415
FFM %	0.34 ± 8.14	–0.65 ± 9.03	0.28 ± 8.81	1.46 ± 7.54	0.492
FFM index	0.57 ± 1.21	0.43 ± 1.49	0.42 ± 1.19	0.54 ± 1.18	0.899

1 Values are presented as mean ± SD unless otherwise indicated. CSB+, corn-soy blend plus; CSWB, corn-soy-whey blend; FFM, fat-free mass; FM, fat mass; MAM, moderate acute malnutrition; MUAC, midupper arm circumference; RUSF, ready-to-use supplementary food; SC + A, super cereal plus amylase; WHZ, weight-for-height *z* score.

2 ANOVA tests used to determine differences among the 4 study arms.

3 Four-week *n* = 225, WHZ; *n* = 218, MUAC; *n* = 205, FFM index.

**FIGURE 3:**
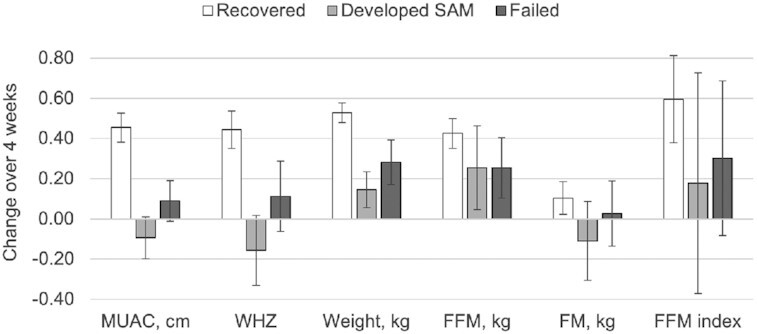
Mean changes in anthropometric and body composition measures in children after 4 wk of treatment for MAM, by study outcome. Error bars represent 95% confidence intervals. Recovered defined as achieving a MUAC ≥12.5 cm (*n* = 149); developed SAM defined as deteriorating to SAM (*n* = 26); failed defined as receiving up to 7 rations but remaining MAM (*n* = 35). Other outcomes not included: lost to follow-up (*n* = 7) and died (*n* = 1). FFM, fat-free mass; FM, fat mass; MAM, moderate acute malnutrition; MUAC, midupper arm circumference; SAM, severe acute malnutrition; WHZ, weight-for-height *z* score.

Changes in body composition varied by study outcome (**Figure 3**); in particular, FM was lost on average only among children who deteriorated to SAM. Children who recovered from MAM (as defined by achieving MUAC ≥12.5 cm within 12 wk) showed some differences in anthropometry and body composition at baseline compared with children who did not recover; they were slightly heavier and had higher MUAC, higher FM, and lower FFM % (**Table 4**). After 4 wk of treatment, children who recovered had larger gains in FFM, FFM index, weight, WHZ, and MUAC (the averages of the latter two did not change at all in the nonrecovered children). There was a similar pattern for FM gain, but it was not significant. Children who recovered and sustained that recovery (defined as maintaining MUAC ≥12.5 cm 4 wk from program exit) had gained more weight, WHZ, and MUAC than children who relapsed, but in this exploratory analysis (i.e., not statistically powered *a priori*), there were no detectable differences in body composition indicators.

**TABLE 4:** Anthropometry and body composition of children at baseline and changes after 4 wk of treatment for MAM, by whether they recovered or did not recover (*n* = 312), and among recovered children, whether they sustained that recovery for 1 mo after program exit or relapsed to MAM (*n* = 219)^1^

	Recovered		Sustained recovery^2^	
Characteristic	Yes	No	Yes	No
*N*	229	83	169	50
Baseline				
Weight, kg	6.62 ± 0.99	6.31 ± 0.85^3^	6.69 ± 1.07	6.37 ± 0.57^3^
WHZ	–1.67 ± 0.73	–1.80 ± 0.76	–1.72 ± 0.74	–1.51 ± 0.68^4^
MUAC, cm	12.0 ± 0.26	11.9 ± 0.25^3^	12.0 ± 0.27	12.1 ± 0.22
Total body water, kg	4.17 ± 0.71	4.10 ± 0.72	4.24 ± 0.74	3.93 ± 0.48^3^
FFM, kg	5.27 ± 0.95	5.17 ± 0.96	5.36 ± 0.99	4.94 ± 0.63^3^
FM, kg	1.35 ± 0.47	1.14 ± 0.48^3^	1.33 ± 0.49	1.43 ± 0.44
FFM %	79.5 ± 6.65	81.7 ± 7.81^3^	80.1 ± 6.61	77.5 ± 6.72^3^
FFM index	11.6 ± 1.09	11.7 ± 1.29	11.6 ± 1.11	11.5 ± 1.07
Change after 4 wk^5^				
Weight, kg	0.53 ± 0.38	0.22 ± 0.33^3^	0.57 ± 0.41	0.38 ± 0.21^3^
WHZ	0.44 ± 0.58	–0.00 ± 0.54^3^	0.49 ± 0.61	0.28 ± 0.44^4^
MUAC, cm	0.45 ± 0.45	0.02 ± 0.32^3^	0.52 ± 0.48	0.25 ± 0.29^3^
Total body water, kg	0.33 ± 0.45	0.18 ± 0.42^3^	0.34 ± 0.46	0.31 ± 0.45
FFM, kg	0.43 ± 0.57	0.24 ± 0.53^3^	0.44 ± 0.58	0.41 ± 0.57
FM, kg	0.10 ± 0.62	–0.02 ± 0.54	0.13 ± 0.64	–0.03 ± 0.55
FFM %	–0.02 ± 8.49	1.02 ± 8.31	–0.34 ± 8.58	1.65 ± 8.48
FFM index	0.60 ± 1.31	0.26 ± 1.21^3^	0.57 ± 1.29	0.67 ± 1.44

1 Values are presented as mean ± SD unless otherwise indicated. Paired *t* tests were used to determine differences between groups. CSB+, corn-soy blend plus; CSWB, corn-soy-whey blend; FFM, fat-free mass; FM, fat mass; MAM, moderate acute malnutrition; MUAC, midupper arm circumference; RUSF, ready-to-use supplementary food; SC + A, super cereal plus amylase; WHZ, weight-for-height *z* score.

2 Defined as recovered children having a MUAC ≥12.5 cm 4 wk after program exit. Ten children were missing follow-up data.

3 Significantly different, *P* < 0.05.

4 Trend, *P* < 0.10.

^5^Four-week *n* = 225, WHZ; *n* = 218, MUAC; *n* = 205, FFM index.

## Discussion

We examined the changes to anthropometry and body composition over 4 wk among children being treated for MAM with 1 of 4 SNFs. Our results showed that FFM comprised the majority of weight gained, and differences in changes to anthropometry and body composition across the study arms were not found to be statistically different. FFM gain in children who recovered was greater than in children who did not recover, and although children who recovered gained FM, children who did not recover did not gain FM, on average.

Our overall body composition results are fairly consistent with previous studies. A study in Burkina Faso evaluated body composition changes using the DD method among 6- to 23-mo-old children with MAM treated for 12 wk with several variants of LNSs or FBFs with different protein sources (21). Their results showed an average baseline body composition of 83.5% FFM, similar to that of our participants (80.1%). At the end of their 12-wk intervention, children had gained an average of 0.90 kg, of which 93.5% was FFM, a higher proportion of FFM compared with the children in our study (82%). A study in Mali also looked at body composition changes among 6- to 35-mo-old children after 12 wk of treatment for MAM with 1 of 4 different SNFs using the DD technique (22). At baseline, children's body composition was 71.4% FFM on average (about 10 percentage points lower than in the Burkina Faso study and our study), and during the intervention, children gained 1.02 kg, 67.6% of which was FFM, a lower proportion of FFM compared with children in our study and in the Burkina Faso study, although the overall change in FFM % was not significant from baseline to endline, similar to our findings. The Mali study also found that recovered children gained more FFM and FM than nonrecovered children, with significantly higher gains in FM%; although a similar pattern was seen in our data for FFM, the differences in FM were not significant (perhaps due to sample size or to the duration between body composition measurement, discussed below). Body composition reference data in well-nourished children show a range of about 32–25% FM from 6 to 24 mo of age, with FM% being slightly higher in girls and declining with age (20). The growth rate for 12- to 18-mo-old girls is 8.1 g/d, of which about 17% is FM and 83% is FFM, a slightly lower proportion of FM than in their existing body composition. Overall, our results are consistent with others in showing a higher proportion of FFM gain compared with FM during treatment for MAM, and the proportion of FM found in children in our study after 4 wk of treatment appears to be slightly less than that of well-nourished populations (20). Newer data that allow standardization of body composition to compare values to reference populations could be useful in future studies (23).

Whereas our study found no differences in anthropometry or body composition changes among the SNFs, both the Burkina Faso (21) and Mali (22) studies did. In Burkina Faso, the children treated with LNS products had significantly larger gains in FFM index, WHZ, and MUAC and a higher recovery rate compared with the children receiving CSB products, and in Mali, the RUSF group gained more FM than children in the Misola (a locally prepared cereal–legume blend with no dairy ingredients) group. The lack of differences among the SNFs in our study could reflect that there is truly no difference in the effects of these particular SNFs on body composition in the context studied or that detectable differences emerge after 4 wk. However, this could also be a type I error due to lower statistical power from a smaller sample size and higher variance than anticipated, or perhaps due to the more nutritionally similar formulations of the SNFs in our study or underlying differences in the populations studied.

One finding of particular interest was that while children who did not recover gained weight during treatment for MAM, on average that weight was almost entirely FFM with little to no FM gained. This finding is at odds with concerns that excess gain in FM is associated with chronic disease risk later in life (7, 8) and even short-term language and motor development (24). Because wasting is associated with decreased FM and may be related to the increased mortality seen with this condition, it would be important that treatment for MAM restore FM (25). A recent review of body composition in children after treatment for wasting indicates that fat mass appears to recover more quickly in the short term, whereas FFM levels may take longer to recover, if ever (26). In either case, whether and to what extent fat mass gain is important to recovery from MAM should be considered in regards to the concern that supplementary foods lead to excess fat gain and the relation to chronic disease risk later in life (12). This FM trend also raises further questions: Why are some children gaining FFM but not FM? How does this affect their recovery from MAM? Could this help identify children at risk of not recovering or relapsing earlier in the MAM treatment process? However, it is important to note that although we saw these trends in the overall group statistics, at an individual level, it was highly variable, with both recovered and nonrecovered children gaining and losing FM and FFM in different amounts and proportions. This could have been a result of the imprecision of the DD method, but it might also be useful to investigate, as mentioned above, what is happening on an individual level when a child is not gaining FM (e.g., if the child has an infection).

We would like to address several limitations to this study. One stems from the lower sample size and higher variance of body composition measures than expected, which decreased statistical power. The high variability/spread of body composition measures persisted even after removing values with known methodologic errors, influential outliers, or extreme biological implausibility. Although we do not currently have reason to suspect systematic bias in the measurements (and thus overall conclusions from the data), they may have been imprecise on an individual level. We attribute this issue mainly to methodologic issues of the DD method in the field. To address these concerns, future studies evaluating body composition in children with MAM could consider sweetening the D_2_O solution. However, the issue of imprecision may well be a limitation to the validity of DD in a field setting, which might be overcome only by newer emerging technologies to measure body composition [e.g., portable bioelectrical impedance instruments (27)]. Another limitation was missing length/height measurements at the 4-wk point, which limited our ability to calculate FFM index to make more standardized comparisons across different heights. Finally, a limitation for comparing our findings to other studies is the length of treatment before body composition follow-up measurements. The 4-wk time point was chosen for our study because children would graduate out of the program after achieving a MUAC of 12.5 cm, and we wanted to ensure children in the study had an equal treatment exposure period. However, our results show that even after only 4 wk of treatment, children who recovered during treatment were already showing differential changes in body composition at 4 wk, consistent with the changes at 12 wk found in previous studies. These changes also appeared to follow a similar pattern among children who recovered but relapsed and those who sustained their recovery. Given the potential differences we are seeing in body composition at baseline and within the first 4 wk of treatment among children with different outcomes, perhaps body composition could be a useful tool to predict how children with MAM will respond to treatment in terms of both recovery and relapse. Predicting relapse would be especially important for identifying children who might need different interventions to achieve sustainable recovery and also for determining program cost-effectiveness when children have to keep being treated repeatedly.

The results of this study contribute to the evidence around body composition changes in young children during treatment for MAM. Although previous studies have found different SNFs to have some differential effects on body composition, our data showed none within the first 4 wk of treatment and little difference in terms of recovery outcomes. Our findings confirm that body composition indicators are not entirely consistent with anthropometric indicators, and further research is needed to understand the extent and relevance of these differences. This is the first study looking at body composition and relapse after treatment for MAM, and although we found potential differences, this needs to be explored further. Questions remain about why some children are gaining different proportions of FM and FFM, whether this is related to recovery and relapse, and whether these children could be targeted in some way to improve their nutritional status beyond current treatment protocols. What is more, it is imperative to determine the qualities of healthy, sustainable recovery, as well as identify and use indicators that accurately assess this (or perhaps, more accurately, predict treatment outcomes). Every child who relapses posttreatment represents a partial programming failure; repeat treatments carry large human and budgetary implications. Aside from the type of SNF used for MAM treatment, environmental or other factors that prevent children from recovering (as well as contribute to malnutrition in the first place) will need to be addressed simultaneously. Further research is needed on all these fronts to ensure that programming aimed at treating MAM and sustaining recovery is as cost-effective as possible.

## Supplementary Material

nxab080_Supplemental_File

## Notes

This work was made possible through support provided by the Bureau for Humanitarian Assistance, formerly the Office of Food for Peace, United States Agency for International Development, under the terms of Contract No. AID-OAA-C-16-00020.

Author disclosures: The authors report no conflicts of interest.

Supplemental Table 1 is available from the “Supplementary data” link in the online posting of the article and from the same link in the online table of contents at https://academic.oup.com/jn/.

Abbreviations used: CSB+, corn-soy blend plus; DD, deuterium dilution; D_2_O, deuterium oxide; FBF, fortified blended food; FFM, fat-free mass; FM, fat mass; LNS, lipid-based nutrient supplement; MAM, moderate acute malnutrition; MUAC, midupper arm circumference; PHU, peripheral health unit; RUSF, ready-to-use supplementary food; SAM, severe acute malnutrition; SC + A, super cereal plus amylase; SNF, specialized nutritious food; TBW, total body water.
