# Supplementary material for: Body Composition Changes in Children during Treatment for Moderate Acute Malnutrition: Findings from a 4-Arm Cluster-Randomized Trial in Sierra Leone
Source: J Nutr. 2021 Apr 20;151(7):2043–50. doi: 10.1093/jn/nxab080 (PMC8245884; doi:10.1093/jn/nxab080)
Supplement: nxab080_Supplemental_File [file nxab080_supplemental_file.docx]

Body composition changes in children during treatment for moderate acute malnutrition: findings from a four-arm cluster-randomized trial in Sierra Leone. Suri, D. et al

**Online Supplemental Material**

Supplemental Table 1: Comparison of baseline characteristics between subjects included in analysis and those who did not complete 4 week measures

|  | **Included in analysis** | **Did not complete 4-week measures** | ***P*-value^1^** |
| --- | --- | --- | --- |
| *n* | 312 | 195 |  |
| Age, months | 12.19 ± 7.18^2^ | 12.41 ± 7.07 | 0.731 |
| Females | 173 (55%)^3^ | 126 (65%) | 0.041 |
| Previous SAM | 78 (25%) | 65 (34%) | 0.041 |
| HAZ | -2.72 ± 1.15 | -2.93 ± 1.46 | 0.077 |
| WHZ | -1.70 ± 0.74 | -1.79 ± 0.70 | 0.215 |
| Wealth quintile |  |  | 0.309 |
| Lowest | 69 (22%) | 31 (16%) |  |
| Low | 52 (17%) | 40 (21%) |  |
| Middle | 62 (20%) | 38 (20%) |  |
| High | 71 (23%) | 41 (21%) |  |
| Highest | 56 (18%) | 44 (23%) |  |

^1^ Determined using T-test for continuous variables and Chi-square test for categorical variables

^2^ Mean ± SD, all such values

^3^ Frequency (percent), all such values
